# Supplementary material for: Investigation of the Effects of Pulse-Atomic Force Nanolithography Parameters on 2.5D Nanostructures’ Morphology
Source: Nanomaterials (Basel). 2022 Dec 11;12(24):4421. doi: 10.3390/nano12244421 (PMC9781517; doi:10.3390/nano12244421)
Supplement: Supplementary file 1 [file nanomaterials-12-04421-s001.zip › nanomaterials-2075082-supplementary.pdf]

SUPPLEMENTARY INFORMATION

Investigation on the effects of the Pulse-Atomic Force Nanolithography parameters on the nanostructures' morphology - a systematic study

Paolo Pellegrino[1,‡], Isabella Farella[2,‡], Mariafrancesca Cascione[1,], Valeria De Matteis[1,], Alessandro P. Bramanti[3], Antonio Della Torre[2], Fabio Quaranta[2] and Rosaria Rinaldi[1,2]

[1] Department of Mathematics and Physics "Ennio De Giorgi", University of Salento, Via Monteroni, 73100 Lecce, Italy.
[2] Institute for Microelectronics and Microsystems (IMM), CNR, Via Monteroni, 73100 Lecce, Italy.
[3] STMicroelectronics S.r.l., System Research and Applications (SRA) Silicon Biotech, Lecce Labs, Via Monteroni, 73100 Lecce, Italy.
‡ The authors contributed equally to this work.
* Correspondence:

In this supplementary, we report the AFM probe deflection (DFL) signals, acquired by the oscilloscope integrated into AFM NTEGRA instrument, relevant to each P-AFL experiment carried out (**Figure S1**, **S3**, and **S4**). In addition, the cross-section profiles of the nanogrooves, patterned by increasing the indentation step values, were shown in Figure **S2**.

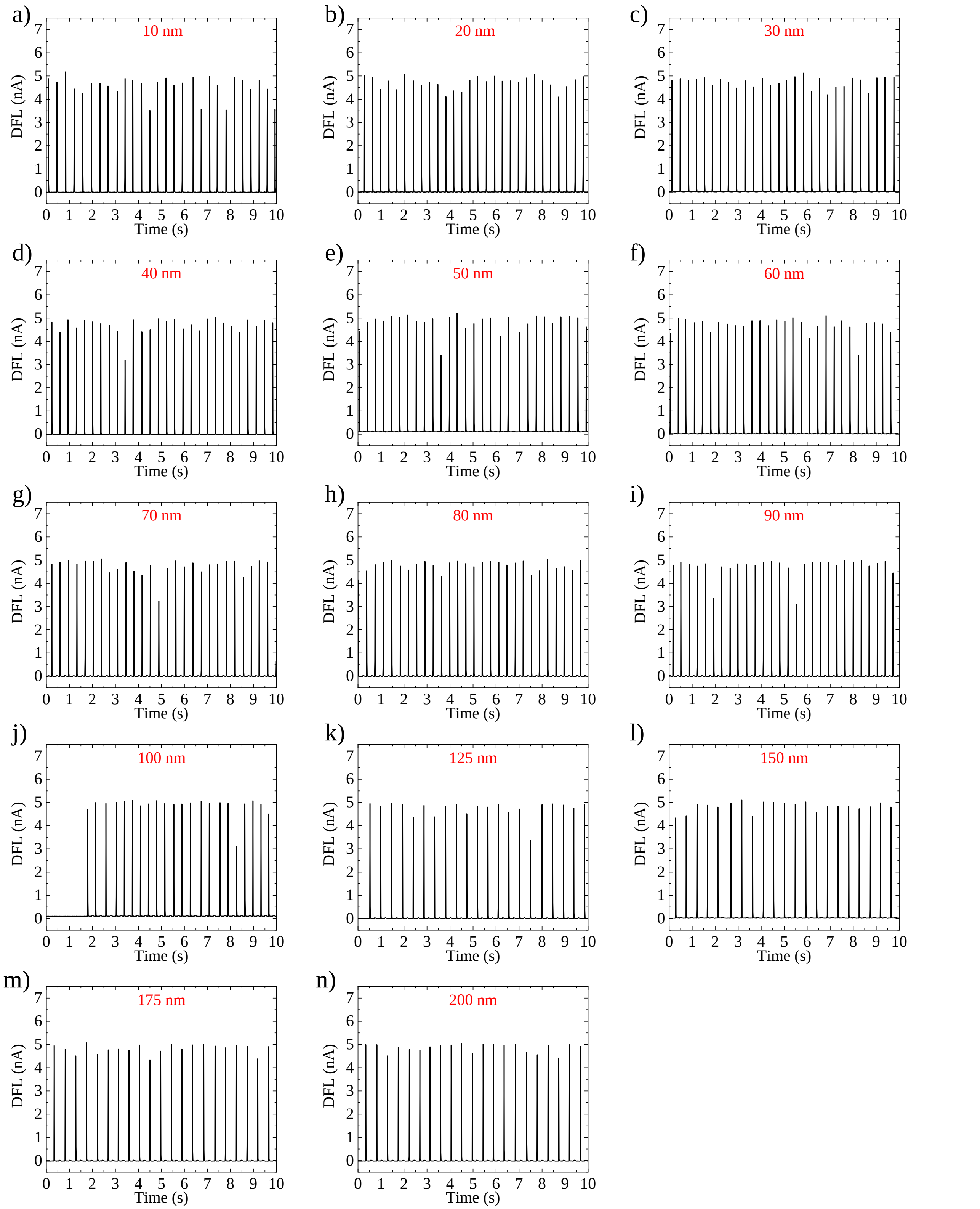

Figure S1. Deflection (DFL) signals acquired during the P-AFL test by means of the oscilloscope, integrated in the AFM NTEGRA instrument, with the aim to investigate the effect of the indentation step on the nanogrooves shape. In these tests, increasing step values, from 10 nm to 200 nm, were used to pattern array of nanogrooves; for each P-AFL test, the corresponding DFL signal was acquired and reported in **Figure S1** (**a**)-(n).

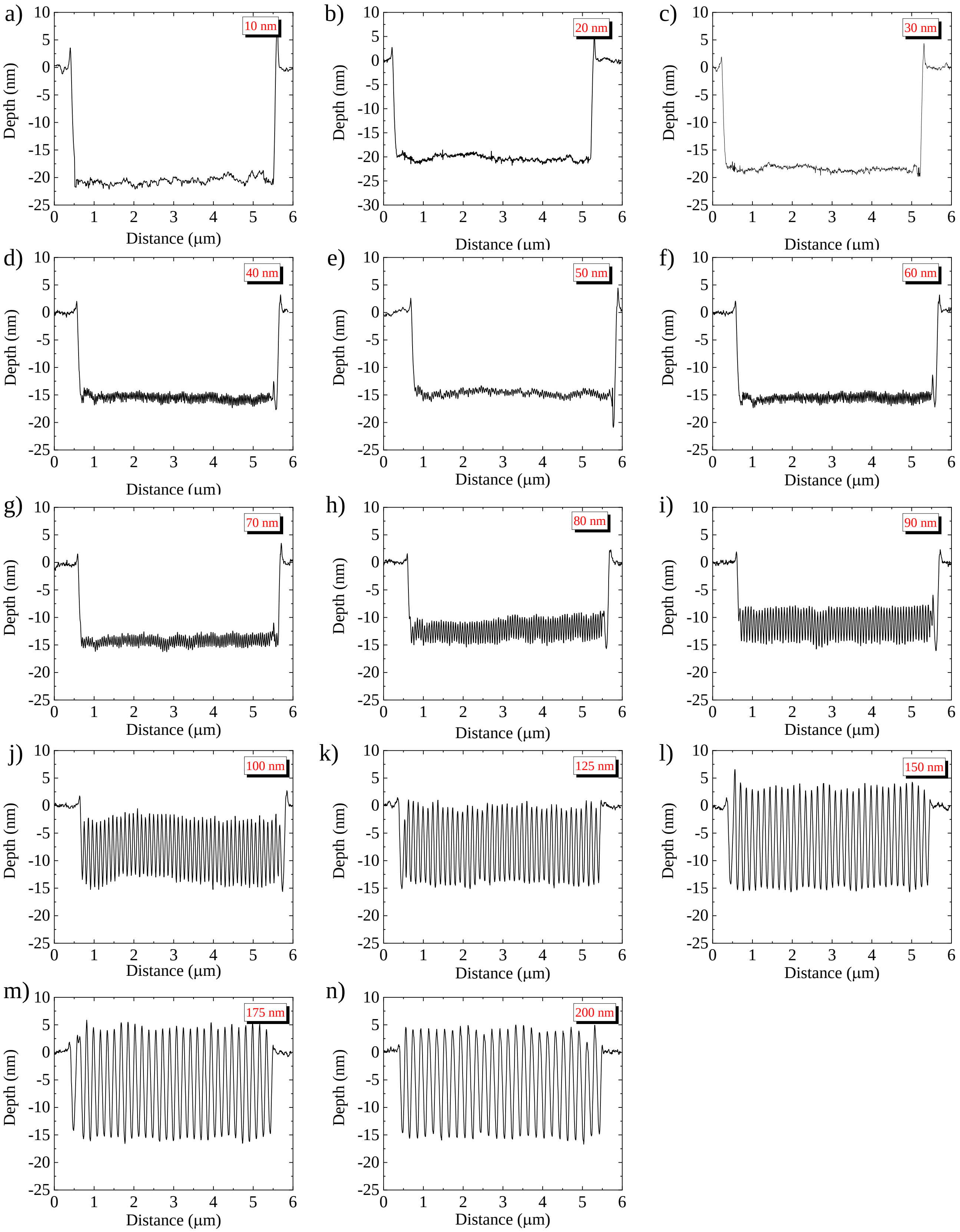

Figure S2. Cross-sections of the nanogrooves patterned by P-AFL, with increasing Step values, from 10 nm to 200 nm. The labels in the graphs indicate the step size used in each test.

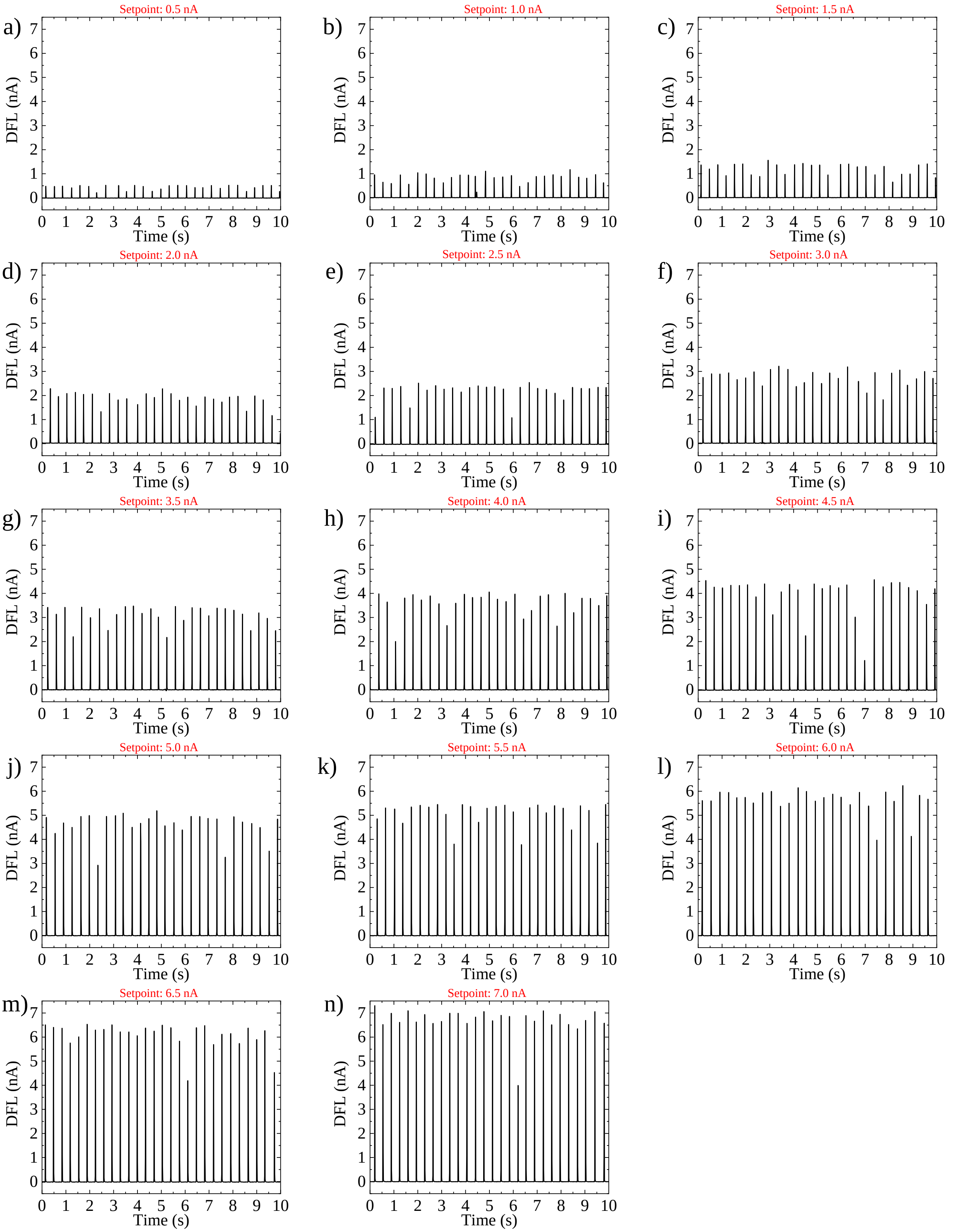

Figure S3. Deflection (DFL) signals of the AFM tip, recorded by the oscilloscope during the P-AFL tests in which the nanogrooves were patterned with an increasing pulse amplitude (Setpoint) from 0.5 nA to 7.0 nA.

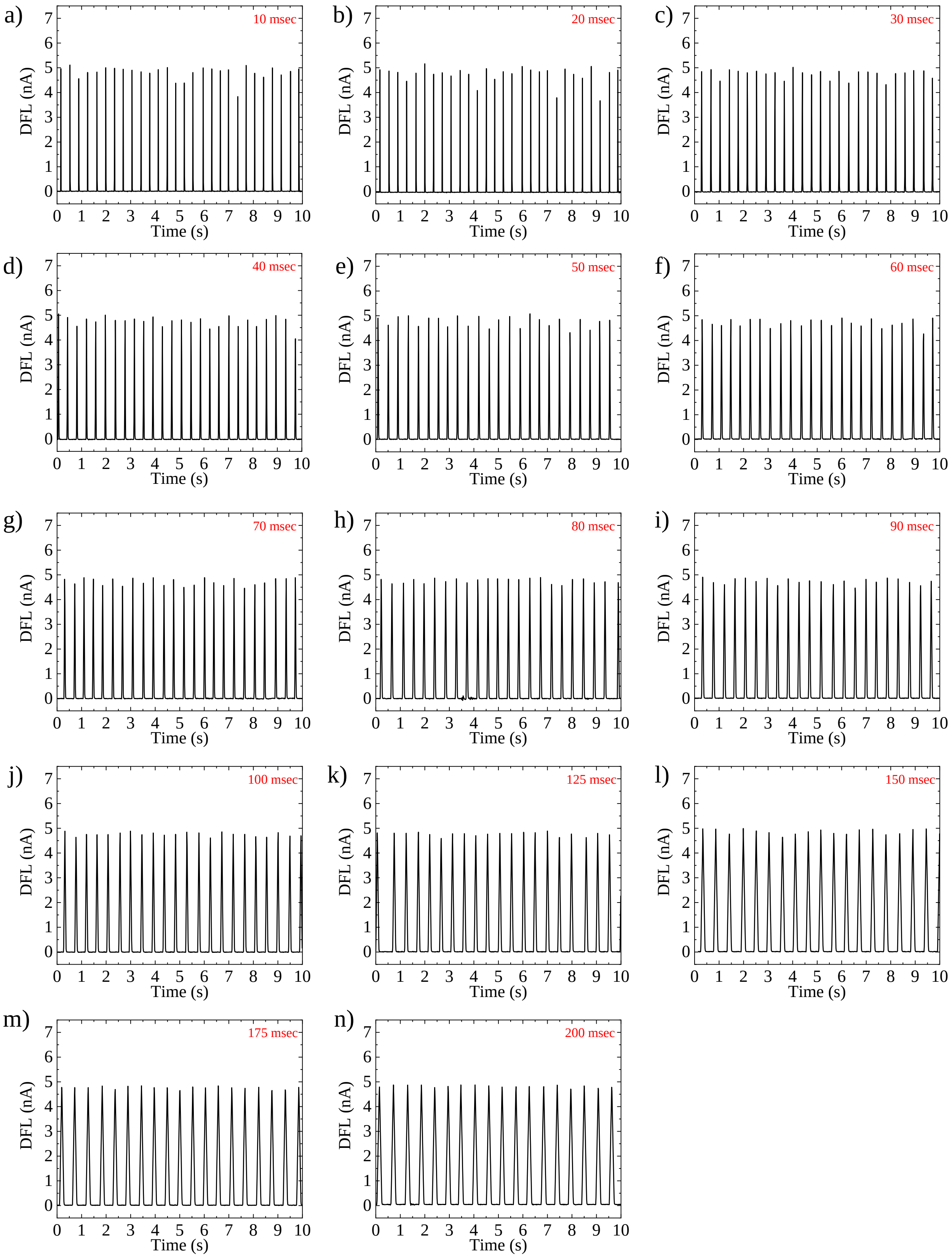

Figure S4. Signals AFM tip deflection (DFL) collected by the oscilloscope in the P-AFL tests carried out to investigate the effect of the Pulse width on the patterned nanogrooves. In each test, an increasing pulse width values from 10 msec to 200 msec were set.
